# Brief Motivational Workplace Intervention to Reduce Sugar-Sweetened Beverage Consumption Among Health Care Workers: Intervention Description, Feasibility, Fidelity, and Exploratory Outcomes From a Cluster Randomized Controlled Trial

**DOI:** 10.2196/96240

**Published:** 2026-10-27

**Authors:** Isabella Fornell-Villalobos, Laurie M Jacobs, Juliana Van Cleve, Kathleen J Antonio, Amanda Pickett, Melody Pezeshkian, Tarini Mudunuri, Ashley E Mason, Jamey M Schmidt, Elissa S Epel, Laura Schmidt, Rachel M Radin

**Affiliations:** 1 Center for Health and Community, Department of Psychiatry and Behavioral Sciences, University of California, 675 18th St, San Francisco, CA, 94107, United States, 1 4158857733; 2 Philip R Lee Institute for Health Policy Studies, University of California, San Francisco, CA, United States; 3 Sutter Health Research Institute, Emeryville, CA, United States; 4 Department of Clinical Psychology, Alliant International University, San Diego, CA, United States; 5 Department of Surgery, University of Miami, Miami, FL, United States; 6 Department of Psychiatry and Behavioral Sciences, Osher Center for Integrative Health, University of California, San Francisco, CA, United States

**Keywords:** sugar-sweetened beverages, brief motivational intervention, cravings, motivational interviewing, diabetes

## Abstract

**Background:**

Metabolic disease, including type 2 diabetes, is a major public health concern. Reducing sugar-sweetened beverage (SSB) consumption is a critical target for disease prevention and lifestyle modification. Workplace interventions offer a promising opportunity to address barriers to behavior change while strengthening motivation and self-efficacy.

**Objective:**

The authors describe the intervention protocol, fidelity, and feasibility of a theory-informed brief intervention based on motivational interviewing principles to support reductions in SSB consumption among health care workers. The authors also explore changes over time in proposed mechanisms of behavior change.

**Methods:**

Participants (N=314) allocated to the intervention arm of a multicenter randomized controlled trial received a 4-dose intervention consisting of an introductory phone call, a brief counseling session, and 2 booster calls, supplemented by at-home tools targeting stress and craving management. We assessed fidelity to motivational interviewing principles (competence scores) and described feasibility via session adherence. Finally, we explored changes over time in motivation for change and perceived self-efficacy.

**Results:**

More than 60% of enrolled participants completed all 4 sessions, suggesting feasibility and high retention in the context of a remotely delivered workplace intervention. Health coaches achieved basic levels of proficiency in 2 of 4 competency scores: technical (mean 3.23, SD 0.48) and complex reflections (mean 41.91%, SD 15.17%). Preliminary exploratory findings indicate improvements in motivation for change and self-efficacy over time. Finally, we found that the use of brief mindfulness practices were associated with acute reductions in stress and cravings.

**Conclusions:**

This theory-grounded brief motivational intervention may offer a cost-effective and accessible approach for employers to support behavior change related to SSB consumption. Findings from the fully powered randomized controlled trial are needed to determine if the brief intervention can effectively change behavior. This paper establishes the intervention and its framework to inform future implementation.

## Introduction

Sugar-sweetened beverages (SSBs) are a major target for obesity and metabolic disease prevention [1]. They are the largest contributor to added sugar intake in the US diet, particularly among lower-income and ethnic minority populations [2,3]. Recommended daily limits for added sugar intake range from 25 to 36 g [4], yet a single 355 mL (12 fluid ounces [fl oz]) soda contains 39 g or more. Daily SSB consumption increases the risk of developing type 2 diabetes by 18% [2] and obesity by 12% [5]. Adults may gain up to 0.45 kg (1 pound) annually for every 236.58 mL (8 oz) of SSB consumed daily, thereby elevating cardiometabolic mortality risk [6].

Sugar cravings and ineffective stress-coping strategies can be major barriers to reducing SSB consumption. Adolescents report more frequent cravings when they stop consuming these beverages [7]. Individuals who eat to relieve stress are more vulnerable to highly palatable sugary options, including SSBs [8]. Sugar activates brain reward pathways that may reinforce habitual intake [9-11], resembling mechanisms observed in substance abuse [12,13]. Combined with the widespread availability and successful marketing of SSBs, stress and cravings may sustain addiction-like consumption patterns that are difficult to disrupt without appropriate intervention [14].

The workplace is a promising environment for intervening in health behaviors. Most adults spend a significant proportion of their waking hours at the workplace and are heavily influenced by its food and beverage environment. SSBs are among the most frequently purchased workplace items [15]. Some institutions have opted to ban SSBs and replace them with healthier alternatives that do not contain added sugar [16-19]. In our pilot study (n=214) [19], a workplace SSB sales ban reduced daily consumption by an average of 243 mL (8.2 fl oz). In combination with a brief motivational intervention (BI), the reduction was 741 mL (25.04 fl oz). Reductions in SSB intake were associated with improvements in insulin sensitivity and reductions in waist circumference, suggesting that employer-sponsored multilevel interventions may effectively promote behavior change and prevent diet-related diseases such as type 2 diabetes.

BIs are short counseling sessions (typically 15‐30 minutes), grounded in motivational interviewing (MI) principles [20,21], that increase individuals’ awareness of the impacts of certain behaviors on short- and long-term health, enhance motivation for change, and build self-efficacy (ie, an individual’s belief in their ability to succeed) [21,22]. These are key elements that support sustained, self-driven behavior change [23]. They are cost-effective and well-established for treating substance use across diverse settings [24-26] and can be effectively delivered by paraprofessionals [27,28]. Telephone or online-based BIs may be effective for SSB reduction, especially when supplemented with booster contacts through text or mobile apps [29-32]. However, these interventions have not been assessed for feasibility; their evaluations often lack rigorous descriptions of the interventions, and they do not explicitly target key mechanisms (eg, stress and cravings) that promote or sustain SSB consumption. In our pilot study, individuals who experienced strong cravings benefited the most from the brief intervention [33].

We developed a BI that incorporates craving and stress management tools and booster contacts for additional support. We are testing the BI in the context of a multilevel randomized controlled trial (RCT), the Workplace Metabolic Health Improvement Study (5R01DK132870-04) [34], which combines a workplace sales ban on SSBs (removal of SSBs from all cafeterias, vending machines, and other workplace sales outlets) with our BI protocol.

This study describes the BI and evaluates its feasibility, as measured by adherence, in a large sample of heavy SSB-consuming health care workers. We draw from MI to inform our BI, which is delivered by health coaches with some graduate-level training in psychology and/or medicine. In addition, we evaluate health coaches’ adherence to MI principles using the Motivational Interviewing Treatment Integrity 4 (MITI 4) coding system [35]. We hypothesized that the BI would be feasible (an adherence rate exceeding 60% at each dose of the intervention) and that health coaches would demonstrate at least a basic level of adherence to MI principles. In a set of exploratory analyses, we examined (1) within-group effects over time in participants’ self-reported motivation for change and self-efficacy, though we did not include a control group; (2) baseline characteristics that predict intervention dropout; and (3) effects of stress- and craving-management tools on changes in self-reported stress, craving, and thirst.

## Methods

### Study Overview

We recruited 3 waves of participants over the course of 9 months and followed each wave for 12 months as part of a 15-site multilevel RCT (n=604). Participants were employed at one of 15 hospital campuses within Sutter Health, a not-for-profit integrated health care system serving Northern California and the Central Coast. Recruitment for wave 1 took place from July 2023 to October 2023, wave 2 from October 2023 to January 2024, and wave 3 from January 2024 to April 2024.

Participants completed baseline online self-report surveys in the 4 to 6 weeks prior to the intervention period. We randomized participants into 1 of 4 conditions: sales ban only, sales ban+BI, BI only, and no sales ban and no BI. In this study, we do not include the entire sample. We report preliminary data from 314 participants in the 2 RCT arms who received the BI: sales ban+BI and BI only.

### Development of the BI

We developed the BI for SSB reduction based on the National Institute on Alcohol Abuse and Alcoholism (NIAAA) standard alcohol brief intervention [36]. We integrated its core elements (eg, providing feedback on the amount of intake), MI principles, and findings from our pilot study (eg, the impact of cravings) [19] to create an intervention that used motivational strategies to reduce SSB consumption.

A trained health coach delivered the intervention via videoconference using a semistructured script that included open-ended questions and reflective listening techniques. The coach tailored feedback and advice by explicitly eliciting participants’ perspectives on how useful they found psychoeducation around SSB consumption and the effects of SSB consumption on short- and long-term health. For example, when participants indicated familiarity with certain content (eg, the consequences of excessive sugar consumption), the coach acknowledged and validated their knowledge and encouraged participants to generate their own motivations for change. Coaches also provided concrete educational elements, such as information on sugar content in common SSBs, to support informed decision-making (eg, “You are reporting you drink XX amount of added sugar per day through XX cans of soda, which exceeds the daily recommended limit by XX amount; What do you make of this discrepancy?”).

Trained health coaches delivered the BI over 6 weeks (in each of the 3 waves). In the protocol structure, we included (1) an initial engagement call, (2) a brief counseling session, and (3) 2 brief booster phone calls. In addition, we granted participants access to a website featuring stress and craving management at-home audio tools (adapted from Mindfulness-Based Stress Reduction and Mindfulness-Based Eating Awareness Training) [37,38].

### Ethical Considerations

This study was approved by the Institutional Review Board (IRB) of Sutter Health (protocol number: 22022.031‐2). All study procedures were conducted in accordance with the ethical standards of the responsible institutional and national committees and the Declaration of Helsinki of the World Medical Association. All participants provided electronic informed consent before engaging in any research activities and were informed that they could withdraw from the study at any time without penalty. Participant privacy and confidentiality were maintained in accordance with the IRB-approved protocol. Study data were deidentified before analysis, and no identifying information is included in this study or Multimedia Appendix 1. Participants received Amazon electronic gift cards totaling US $60 for completing study activities.

### Participants

Participants were full-time employees at one of 15 affiliated health care centers within Sutter Health—a not-for-profit integrated health care system serving Northern California and the Central Coast—and reported consuming at least 3 SSBs per week (~12‐14 g of added sugar per day). Health care workers may particularly benefit from the BI to reduce SSB consumption, as they face chronic stress due to long shifts, irregular working hours, and high job demands [39]. Exclusion criteria included non-English speaking and pregnancy.

### Brief Intervention Procedures

#### Brief Intervention Protocol

The intervention consisted of 4 doses delivered over a 6-week period, supplemented by continuous access to stress- and craving-management tools. The target delivery timing and the observed delivery window are summarized in Table 1.

##### Initial Engagement Phone Call

The intervention began with a brief (10‐ to 15-minute) phone call to engage participants. The goal was to provide an overview of the program, explain its rationale, focus on SSB intake, and “plant seeds of change [20]” (Appendix 1 in Multimedia Appendix 1).

**Table 1.** Timing and content of the brief intervention.

Intervention component	Target delivery timing	Observed delivery timing (days)
Dose 1: initial engagement phone call	Week 1	—^a^
Dose 2: counseling session	~1 wk after dose 1	4‐10^b^
Stress and craving management tools	Continuous access	—
Dose 3: booster call 1	~1 wk after dose 2	7‐10^b^
Dose 4: booster call 2	~3 wk after dose 3	21‐24^b^

a Not applicable.

b Observed delivery windows represent the intervals during which the greatest concentration of participants received each subsequent intervention dose, based on actual delivery dates. These windows are descriptive of timing intervention delivery and accommodate participants’ availability.

##### Brief Counseling Session

A week after the initial phone call, the health coach followed a semistructured protocol via a secure, telehealth video visit or phone call to (1) engage participants in a conversation about any concerns regarding their level of SSB consumption; (2) encourage reflection on why and how reducing SSB intake may be important or in line with their values; (3) identify motivational factors for changing behavior; (4) with permission, present educational guidelines around sugar consumption and its health and metabolic impacts; (5) explore perceived barriers to reducing SSB intake and discuss alternative solutions; (6) collaborate on specific, achievable goals; (7) assess self-efficacy and perceived importance of change; and (8) introduce the study website as a resource containing audio tools designed to help manage stress, cravings, and thirst. Sessions lasted between 25 and 30 minutes on average. If participants consented, sessions were audio-recorded for quality control and treatment fidelity assessment (Appendix 1 in Multimedia Appendix 1).

##### Booster Calls

One week after the brief counseling session, the health coach followed a semistructured protocol via a phone call to (1) review participants’ progress toward their specific goal(s) and reflect upon and affirm any successes, (2) identify challenges toward their goal(s) and problem-solve or reevaluate, (3) reassess motivational factors for behavior change (“why is this still important to you?”), (4) identify supportive behaviors that promote change (eg, sugar-free replacements), (5) collaborate on next steps for continued behavior change, and (6) reassess perceived self-efficacy and motivation to reduce SSB intake. Approximately 2 weeks later, the health coach followed a similar protocol as in the first booster call. Each booster call lasted 10 to 15 minutes. In the second booster call, which was the final point of contact, the health coach also encouraged participants to reflect on their overall progress and what lessons or tools they would like to take with them once the study ended (Appendix 1 in Multimedia Appendix 1).

##### At-Home Audio Tools

During the counseling session, the health coach introduced participants to a study website that provided general information and brief audio exercises (3‐7 minutes each) on stress management and craving management. Participants were encouraged to access these online tools at least once per week. The health coach asked participants about the frequency of website usage during each booster call. Access to the website was anonymous to protect participant privacy. Therefore, data on website access and usage could not be linked to individual participants, and usage data are presented in aggregated form (Appendix 2 Multimedia Appendix 1).

##### Educational Materials

During the counseling session, the health coach presented a brief PowerPoint (Microsoft) slide deck containing educational material that covered (1) the effects of excessive sugar consumption on the body (eg, risk of tooth decay, type 2 diabetes, and liver disease); (2) American Heart Association guidelines on the daily recommended amount of added sugar vs average added sugar amounts in the typical American diet; (3) amounts of added sugar (in grams) in typical SSBs; and (4) alternatives to sugary beverages (eg, sparkling water and unsweetened iced tea). Materials were adapted from SugarScience [21,40], a source on the scientific evidence of sugar’s impact on health developed by health scientists from the University of California, San Francisco (Appendix 3 in Multimedia Appendix 1).

### Health Coach Training and Fidelity of Brief Intervention Delivery

We recruited graduate-level psychology trainees, as well as medical school graduates, with at least 1 to 2 years of clinical experience, to serve as health coaches for this study. Health coaches first completed a 6-week training course on basic MI principles and skills, led by a member of the Motivational Interviewing Network of Trainers (MINT), Inc, an American Association for Marriage and Family Therapy (AAMFT)–approved supervisor, and a California Association of Marriage and Family Therapists (CAMFT)–approved provider, with more than 10 years of experience in MI training and supervision. Training sessions included discussions about key concepts, mock sessions, and role-plays (Appendix 4 in Multimedia Appendix 1). Once health coaches were deemed competent by the study’s clinical supervisor to deliver the BI, they continued to receive ongoing weekly supervision with a licensed clinical psychologist. The supervisor reviewed audio-recorded counseling sessions and provided detailed written and verbal feedback to health coaches (Appendix 4 in Multimedia Appendix 1).

### Measures

#### Intervention Feasibility

To determine the feasibility of the BI, we examined retention rates across all study waves and intervention doses: the initial engagement phone call (dose 1), counseling session (dose 2), and booster calls 1 and 2 (doses 3 and 4, respectively).

#### Fidelity to MI Principles

We used the MITI scoring version 4.2 [35] to assess fidelity to MI principles during the brief counseling session. The MITI 4.2 includes 4 competence thresholds: technical global, relational global, reflection-to-question ratio (R:Q), and percentage of complex reflections. These competence scores are calculated based on 4 global ratings and 10 behavior counts (Table S1 in Multimedia Appendix 1). All 14 items focus on MI practitioner skills and behaviors (and not on participant behaviors/responses). The 4 global ratings require the coder to assign a single number from a 5-point scale to assess a 20-minute excerpt of a session (eg, empathy and cultivating change talk). Each global rating score is meant to capture an overall judgment about the dimension being evaluated. A behavior count requires the coder to tally instances of interviewer behaviors (eg, questions and reflections). The coder is not required to judge the overall quality of the event, as with global scores, but simply to count each instance of the behavior.

Across 3 study waves, 247 sessions were eligible for video recording, of which 199 were available for coding and constituted the sampling denominator for the MITI 4.2 scoring. We randomly selected approximately 20% (n=41, 20.6%) of the available recordings within each wave for independent coding to ensure representation across all 3 study waves. In line with previous studies, of the 41 independently coded recordings, we randomly selected approximately 20% [41] (n=8, 19.5%) to calculate interrater reliability using intraclass correlation coefficients (ICCs).

To generate the random samples for independent coding (n=41) and interrater reliability (n=8), we assigned each recording a random number between 0 and 1 using the RAND() function in Microsoft Excel. We then sorted the recordings in descending order by the assigned random numbers. Then, we selected recordings sequentially from the top of the randomized list until we reached the predetermined sample size.

We were unable to ensure equal representation among health coaches because of personnel turnover and variability in coach availability. Therefore, participants were assigned based primarily on coach availability. Table 2 shows the distribution of health coaches represented in the randomly selected sample of 41 recordings.

**Table 2.** Random distribution of health coaches represented in the random sample of recordings (n=41).

Health coach	Recordings, n (%)
Health coach 1	7 (17.1)
Health coach 2	5 (12.2)
Health coach 3	5 (12.2)
Health coach 4	4 (9.8)
Health coach 5	4 (9.8)
Health coach 6	3 (7.3)
Health coach 7	3 (7.3)
Health coach 8	3 (7.3)
Health coach 9	2 (4.9)
Health coach 10	2 (4.9)
Health coach 11	1 (2.4)
Health coach 12	1 (2.4)
Health coach 13	1 (2.4)

#### Self-Reported Ratings of Motivation for Change and Perceived Self-Efficacy

Throughout the intervention, the health coach asked the participant to rate their motivation for change and their perceived self-efficacy to make that change (eg, reducing SSB intake) at time points: (1) during the counseling session, (2) booster call 1, and (3) booster call 2.

To evaluate motivation for change, the health coach used the “importance strategy” [20], which evokes motivation for change by asking, “On a scale from 1 (not at all interested) to 10 (most interested), how interested are you in reducing the number of sugary drinks you consume? In other words, how important would you say ______ is for you to ______ (eg, reduce SSB intake) right now?” To encourage deeper reflection, the coach follows up with, “And why are you at a ______ and not a lower number?” The goal is for the participants to advocate for their own change, while the counselor reinforces their need for change by reflecting it back to them.

Similarly, the health coach used the “confidence ruler” strategy [20] to assess and strengthen perceived self-efficacy. This strategy involves asking, “Now that we have identified some new ideas, how confident are you that you can meet your goal of ______ on a scale from 1 (not at all) to 10 (extremely confident)? (reiterate the participant’s goal/plan regarding SSB intake).” This question should be followed by an open-ended question, such as “What would it take for you to go from ______ (low number) to ______ (high number)?” The aim is to evoke personalized strategies for success. The counselor reinforces self-efficacy by reflecting what the participant has said.

#### At-Home Audio Tools: Self-Reported Stress, Cravings, and Thirst

We prompted participants to rate their levels of stress, cravings, and thirst on a scale from 1 to 10 before and after each at-home audio tool.

### Data Analysis

We conducted statistical analyses using SPSS (version 29.0.2; IBM Corp) and Stata (StataCorp LLC). We computed descriptive statistics to summarize baseline characteristics of the sample. To assess adherence to the intervention, we calculated the percentage of participants retained at each dose. To evaluate health coach fidelity to MI principles, we calculated means, SDs, medians, and ICCs for the 4 threshold competence scores, the 4 global ratings, and the 10-behavior count.

We tested normality before conducting a repeated-measures ANOVA to explore changes in motivation for change and perceived self-efficacy across 3 time points (counseling session, booster 1, and booster 2). Both variables violated normality, so we applied a log2 transformation to meet parametric assumptions. Mauchly test indicated sphericity violations for motivation (*χ*²_2_=77.0; *P*<.001) and self-efficacy (*χ*²_2_=6.5; *P*=.04); Greenhouse-Geisser corrections were applied (ε=0.758 and ε=0.969, respectively). For clarity, results are reported on the original 1 to 10 scale.

We constructed linear regression models to explore whether there were any baseline factors that predicted treatment adherence (number of sessions attended), change in self-efficacy, and change in importance. In the case of each of these outcomes, we examined age, gender, race/ethnicity, BMI, cravings, reward-based eating, and baseline SSB consumption. For models predicting change in self-efficacy and change in importance, we included baseline BMI and the baseline levels of each outcome (in line with standard practice for modeling change scores as outcomes). To explore changes in stress, cravings, and thirst following the audio exercises, we conducted a series of 2-tailed paired-samples *t* tests.

## Results

### Participant Characteristics at Baseline

Table 3 describes baseline sample characteristics. The majority (n=247, 78.7%) of participants (age: mean 41.04, SD 10.52 years) identified as female. Most participants identified as non-Hispanic White (n=138, 43.9%). BMI was in the overweight range (mean 29.63, SD 6.98 kg/m^2^), and participants reported consuming approximately 976 mL (33 fl oz) of SSBs per day (~three standard 354.8 mL [12 fl oz] soda cans per day).

**Table 3.** Baseline sample characteristics (N=314)^a^.

Characteristics	Values
Age (y), mean (SD)	41.04 (10.52)
Craving scale score, mean (SD)^b^	19.71 (16.0)
SSBs^c^ in mL/day^d^, mean (SD)	967.6 (855.58)
Sex, female, n (%)	247 (78.7)
RED^e^, mean (SD)^f^	22.99 (11.17)
BMI, mean (SD)	28.64 (6.98)
Ethnicity, n (%)	
Non-Hispanic White	138 (43.9)
Non-Hispanic Black	14 (4.5)
Non-Hispanic Asian	72 (22.9)
Hispanic	72 (22.9)
Other/unknown	18 (5.7)

a Sample size varied across variables due to missing or unreported values (age: n=311; SSB/day: n=303; sex: n=310; RED: n=300; and BMI: n=312).

b Craving scores ranged from 0 to 75, with higher values indicating greater craving.

c SSBs: sugar-sweetened beverages.

d Equivalent mean in fluid ounces is 32.72 (SD 28.93) per day.

e RED: reward-based eating.

f RED scores ranged from 0 to 52, with higher values indicating more preoccupation with a perceived lack of control over overeating control and a lack of satiety.

### Intervention Feasibility

We enrolled 314 health care workers in the BI across the 3 study waves. The majority (n=276, 87.9%) completed the introductory phone call. Similarly, 78.7% (n=247) of participants completed the counseling session, 71.0% (n=223) completed booster call 1, and 65.9% (n=207) completed booster call 2. A total of 27.7% (n=87) of enrolled participants were lost to follow-up after 3 unsuccessful contact attempts, and 6.7% (n=20) dropped out of the intervention. All reported numbers reflect data aggregated across all 3 waves of the intervention. A summary of findings is presented in Table 4.

**Table 4.** Participant retention throughout all doses of the brief intervention (N=314).

Intervention dose	Noncompleters, n (%)		Total completers, n (%)
	Nonresponders^a^	Dropouts^b^	
Initial engagement phone call	32 (10.2)	6 (1.9)	276 (87.9)
Brief counseling session	22 (7.0)	7 (2.2)	247 (78.7)
Booster call 1	19 (6.1)	5 (1.6)	223 (71.0)
Booster call 2	14 (4.5)	2 (0.6)	207 (65.9)

a Participants were considered as nonresponders after 3 unsuccessful attempts to schedule or complete the intervention doses.

b Participants were classified as dropouts if they withdrew verbally from either the intervention or the study.

### Fidelity to MI Principles

Table 5 presents individual mean and median threshold competence scores for a 20% (n=41 sessions) random sample of recorded BI sessions. These were assessed by 2 blinded, independent MITI coders for adherence to the principles of MI. Health coaches achieved “fair” ratings on 2 categories: technical (mean 3.23, SD 0.48) and percentage of complex reflections (mean 41.91%, SD 15.17%). Health coaches achieved lower ratings for the remaining 2 categories: relational (mean 3.08, SD 0.63) and ratio of reflections to questions (mean 0.79, SD 0.93). Health coaches frequently engaged in “giving information,” which resulted in the highest number of behavior counts (mean 7, SD 3.55) across all categories. Significant ICC values ranged from 0.77 to 1.000 indicating good to excellent reliability for half of the measures assessed. ICC results for the remaining 10 outcome measures are presented in Table S1 in Multimedia Appendix 1.

**Table 5.** Fidelity to motivational interviewing (MI) principles (n=41).

Competence category	Mean (SD)	Median (IQR)	Intraclass correlation
Relational global^a^ (benchmark 3.5‐4)	3.08 (0.63)	3.00 (2.50-3.50)	0.816^b^
Partnership	3.10 (0.70)	3.00 (3.00-3.50)	0.500
Empathy	3.07 (0.68)	3.00 (3.00-3.00)	1.000
Technical global^c^ (benchmark 3‐4)	3.23 (0.48)	3.50 (3.00-3.50)	0.873^b^
Cultivating change talk	2.83 (0.66)	3.00 (2.00-3.00)	0.774^b^
Softening sustain talk	3.63 (0.53)	4.00 (3.00-4.00)	−0.167
Ratio of reflections to questions^d^ (benchmark 1:1-2:1)	0.79 (0.93)	0.65 (0.52-0.76)	0.482
Complex reflections^e^ (%), (benchmark 40‐50)	41.91 (15.17)	41.66 (33.0-48.5)	0.445
Total MI adherence^f,g^	3.49	3.00	—^h^
Total MI nonadherence^i,j^	0.96	1.00	—

a Average between partnership and empathy scores.

b n=8.

c Average between cultivating change talk and softening sustain talk scores.

d Total reflections score divided by total questions score.

e Complex reflection score divided by total questions score.

f Total score calculated as the sum of seeking collaboration, affirm, and emphasizing autonomy scores.

g SD and IQR values were not applicable for MI-adherent behavior score.

h Not applicable.

i Total score calculated as the sum of persuading and confronting scores.

j SD and IQR values were not applicable for MI-nonadherent behavior score.

### Changes in Self-Reported Motivation for Change and Self-Efficacy

We found significant main effects for changes in motivation (*F*_1.52,304.65_=32.21; *P*<.001) and self-efficacy (*F*_1.94,391.42_=7.60; *P*<.001) over time using log-transformed values. This pattern of results was consistent with the analysis on the untransformed data. For ease of interpretation, we report values using the original 1 to 10 scale.

We found a significant increase in motivation for change from the counseling session (mean 7.52, SD 2.34) to booster call 1 (mean 8.35, SD 1.73; *P*<.001), and similarly from booster call 1 to booster call 2 (mean 8.72, SD 1.60; *P*=.03). We also found a significant increase in self-efficacy between the first booster call (mean 7.99, SD 1.73) and the second booster call (mean 8.40, SD 1.55; *P*=.006).

We used linear regression models to examine baseline characteristics that may predict adherence to the intervention, measured by the number of completed doses, as well as changes in motivation and self-efficacy. After adjusting for baseline measures of each dependent variable, Hispanic participants had lower adherence to the intervention compared with non-Hispanic White participants (β=–.35, SE=.13; *P*=.009). Black participants also had lower adherence to the intervention compared with non-Hispanic White participants (β=–.82, SE=.27; *P*=.003); however, this finding should be interpreted cautiously because the sample of Black participants was small (n=14). Moreover, we found that higher baseline BMI was associated with lower self-efficacy ratings over time (β=–.04, SE=.017; *P*=.02). No baseline factors predicted motivation for change over time.

### Participant Engagement With Online Mindfulness-Based Tools

Participants anonymously accessed a resource website with mindfulness-based tools for stress and craving management 121 times during the 12-month observation period. These accesses represented single sessions of website use, with each session including paired pre-audio and post-audio ratings. After listening to an introductory stress management audio, participants reported a significant reduction in self-reported stress (*t*_46_=4.14; *P*<.001), cravings (*t*_45_=3.08; *P*=.004), and thirst (*t*_43_=3.29; *P*=.002; Table 6). Similarly, after listening to an introductory craving management audio, participants showed statistically significant decreases in self-reported stress (*t*_29_=3.24; *P*=.003) and thirst (*t*_28_=3.64; *P*=.001). Listening to the daily stress management practice also resulted in significant reductions in stress (*t*_13_=3.58; *P*<.001) and thirst (*t*_36_=2.89; *P*=.006). Finally, the daily craving management practice led to a significant decrease in self-reported cravings (*t*_13_=2.41; *P*=.03).

**Table 6.** Pre-post changes in self-reported stress, cravings, and thirst.

Mindfulness audio modules	Pre-changes, mean (SD)	Post-changes, mean (SD)	*t* test (*df*)	*P* value
Stress management audio (introductory)				
Stress	4.43 (2.56)	3.28 (2.44)	4.14 (46)	<.001
Craving	4.04 (2.88)	3.13 (2.40)	3.08 (45)	.004
Thirst	4.59 (2.37)	3.68 (2.45)	3.29 (43)	.002
Craving management audio (introductory)				
Stress	3.60 (2.64)	3.07 (2.62)	3.24 (29)	.003
Craving	5.25 (2.66)	5.72 (1.23)	−0.218 (31)	.83
Thirst	4.72 (1.68)	3.66 (2.40)	3.64 (28)	.001
Stress management (daily practice)				
Stress	3.78 (2.63)	2.89 (2.65)	3.58 (13)	<.001
Craving	3.64 (2.58)	2.89 (2.45)	1.73 (35)	.09
Thirst	4.05 (2.46)	2.89 (2.42)	2.89 (36)	.006
Craving management (daily practice)				
Stress	3.67 (1.96)	3.26 (1.83)	1.73 (15)	.10
Craving	4.47 (2.5)	3.40 (1.88)	2.41 (13)	.03
Thirst	4.50 (2.65)	3.71 (2.33)	2.06 (13)	.06
Additional practice (fullness awareness)				
Thirst	3.71 (2.39)	3.19 (2.25)	1.25 (20)	.22

## Discussion

### Principal Findings

We described and evaluated a workplace BI that targeted reductions in SSB intake in a sample of 314 employees assigned to the intervention arm of a multisite RCT in a large health care system. We adapted the BI from the NIAAA standard alcohol brief intervention and revised it to align more closely with MI principles. Trained health coaches delivered the BI remotely over 6 weeks, and participants spent approximately 1 hour in total in scheduled contact. We evaluated motivation for change and perceived self-efficacy throughout the intervention period. Finally, we identified baseline characteristics that predicted intervention dropout and changes in self-efficacy. Building on the effects reported in our pilot study [19,33], this paper provides a detailed protocol, making it available for implementation by other practitioners.

Our BI demonstrated strong retention, with 65.9% (207/314) of participants completing the full intervention and 87.9% (276/314) completing its core component (the brief, 25-min counseling session). These rates compare favorably with the mean adherence rate of 45% reported in a meta-analysis of workplace counseling interventions [42]. Most attrition occurred before the introductory phone call (dose 1), followed by the counseling session (dose 2). We observed small but significant adherence differences by race and ethnicity. Hispanic participants completed fewer sessions than non-Hispanic White participants after adjusting for covariates. Black participants showed a similar pattern, although we interpreted this finding cautiously given the small sample of Black participants (n=14). Prior studies report lower adherence by minority groups in remote health programs, including lower participation in voice-based contacts rather than SMS in remote diabetes management programs [43]. Work on cultural adaptation has shown that tailoring interventions may improve retention [44]. Hispanic participants reported that acknowledging immigration background, family dynamics, and community context supported engagement [45]. Future work-based interventions should recruit larger, more diverse samples and test culturally responsive adaptations to improve treatment acceptability, retention, and generalizability.

To evaluate adherence to MI principles by the health coaches, 2 independent coders assessed 20% of the recorded counseling sessions. Coaches used MI adherent behaviors much more often than nonadherent behaviors. Fidelity on the technical global dimension and the percentage of complex reflections partially supported our hypothesis that coaches would exceed basic competence thresholds. We intentionally included educational content about sugar consumption within the counseling session, in line with the NIAAA brief intervention framework of information delivery. Across the 41 coded sessions, giving information occurred on average 7 (SD 3.55, range: 2‐17) times per session. This design choice may have reduced opportunities for MI techniques and lowered the relational global and reflection-to-question scores [35]. Supporting this possibility, higher giving-information frequency was significantly associated with lower relational global scores (*r*=−0.531; *P*<.001) and a lower R:Q ratio (*r*=−0.264; *P*=.10), although it did not reach statistical significance. These findings suggest a potential tension between giving information and maintaining relational aspects of MI. This tension may be reduced by strengthening training on how to deliver key information within an MI and relational framework.

Among intervention completers with nonmissing data, participants reported significant increases in motivation for change and self-efficacy over time. Although these changes are consistent with the proposed mechanism of MI-based behavior change, they should be interpreted as exploratory and with caution given the absence of a non-BI comparison group. In our pilot study, participants with stronger baseline craving benefited the most from the BI [33]. Given the highly rewarding nature of sugar and its role in reinforcing dopamine-driven craving [8,10], habitual SSB consumption may disrupt reward, pleasure, and stress systems. Enhancing motivation for change and self-efficacy may help counteract these disruptions. By eliciting “change talk” (a key component in MI) and supporting individual goal setting, our BI may empower individuals to develop their own strategies to reduce SSB intake and improve long-term metabolic health.

We also observed clinically relevant heterogeneity by baseline BMI. Prior studies have found that a higher BMI is associated with lower eating self-efficacy and greater difficulty losing weight [46]. Self-efficacy enables individuals to adopt and sustain dietary changes [47]. Consistent with these findings, we found that individuals with higher BMI reported lower confidence in making changes by the end of the intervention. Improvements in self-efficacy may be associated with weight loss [48], and when participants did not observe weight change during the BI window, they may have interpreted this as a lack of progress. Future iterations could add targeted support and education that emphasizes the benefits of SSB reduction beyond short-term weight change, while providing alternative progress indicators.

### Limitations

We assessed motivation for change and self-efficacy using the “importance ruler” and the “confidence ruler” strategies, 2 tools commonly used in MI to strengthen motivation [20]. Although these tools are valuable in clinical practice, they can increase susceptibility to demand characteristics in a research context. Participants may shape responses to align with perceived coach expectations or to maintain rapport [49]. Therefore, incorporating validated questionnaires to assess trait-level motivation and self-efficacy would be important for future studies of this intervention. Another limitation related to our exploratory analyses is that we could not determine whether multiple website–use sessions originated from the same participant. Although pre-audio and post-audio ratings were linked within each session, potential dependence across sessions could not be accounted for. Therefore, future studies should systematically collect information that allows for the identification of unique participants.

An advantage of BIs is that they can be delivered by paraprofessionals without requiring extensive training or formal credentialing. Prior studies showed that probation officers [27] and college counselors [28] successfully delivered BIs. In this study, aspiring counselors and medical providers reached a basic level of competence on 2 metrics of MI competence. Based on the results of our pilot study, it is likely that this level of proficiency supported meaningful behavior change. Results from the RCT will clarify whether this is the case.

### Conclusions

This new workplace BI, part of the Metabolic Health Improvement Program, offers a cost-effective, accessible approach for employers to support reductions in SSB intake among employees. The BI targets proposed mechanisms of change and appears feasible for delivery in large workplace settings. However, our exploratory findings related to the proposed mechanisms of change do not establish intervention effects and require confirmation in the fully powered RCT. Our analysis found high levels of adherence by employees and fidelity to technical MI principles among trained health coaches. An important advantage of BI is its value for populations that might not otherwise seek preventive health services due to limited time and awareness. The inclusion of stress and craving management tools in this BI not only has the potential to support reductions in SSB intake but also may contribute to broader improvements in well-being.

## Supplementary material

10.2196/96240 Multimedia Appendix 1 Protocol training materials and additional results.

## Abbreviations

AAMFT: American Association for Marriage and Family Therapy
BI: brief motivational intervention
CAMFT: California Association of Marriage and Family Therapists
fl oz: fluid ounce
ICC: intraclass correlation coefficient
IRB: Institutional Review Board
MI: motivational interviewing
MINT: Motivational Interviewing Network of Trainers
MITI 4: Motivational Interviewing Treatment Integrity 4
NIAAA: National Institute on Alcohol Abuse and Alcoholism
R:Q: reflection-to-question
RCT: randomized controlled trial
SSB: sugar-sweetened beverage
