# Supplementary material for: Brief Motivational Workplace Intervention to Reduce Sugar-Sweetened Beverage Consumption Among Health Care Workers: Intervention Description, Feasibility, Fidelity, and Exploratory Outcomes From a Cluster Randomized Controlled Trial
Source: JMIR Form Res. 2026 Oct 27;10:e96240. doi: 10.2196/96240 (PMC13717331; doi:10.2196/96240)
Supplement: Supplementary file 1 [file formative-v10-e96240-s001.docx]

## Multimedia Appendix 1

### Appendix 1: Sample script and BI protocol

#### Introductory phone call script

Hello, am I speaking with _____?

(If yes) Hi, my name is _____ and I am a health coach with Sweet Study.

I’m calling to let you know that you have been assigned to the health coaching group.

Do you have a few minutes to speak about the Health Coaching program and schedule your 25-minute coaching meeting?

(If yes) Good. First, I’ll walk you through what is involved and answer any questions. Then, we can schedule your session. Are you in a place where you have some privacy?

(If no) Okay, what’s a better time to speak for 5 minutes, ideally later today? (or tomorrow?)

*Introducing coaching session:* There’s one coaching meeting involved- this is a 15-to-25-minute online meeting with a health coach (me) and will be scheduled–we aim to be flexible based on your work schedule. The meeting might be recorded for quality control and to make sure I’m asking the right questions. But all of your information will be kept confidential, and no one will have access to the information you provide during the meeting other than the study team.

Let’s find a good day that works for you in the next week. I have the following days open: ________________. What day(s) works best for you? What time(s) works best for you?

- *Schedule ideally within the next week (but 2 weeks max)*
- *Ask for top 3 day/time preferences and have them rank-order their preference.*

Once we end this call, I will send you a confirmation email with the link to your 25-minute meeting with me, your health coach. After this meeting, I will call you twice- these are brief 5 to 10-minute calls to check in on your health goals- over the course of 4 weeks. Ok, so that’s the general overview of how this works. Any questions for me before I give you some details about the program itself?

*Introducing program specifics*

Now we’ll go into what this program involves (the specifics) and why we’re doing it. As the focus of the overall study is on metabolic health, one very important factor affecting our health is how much sugary drinks we drink on a daily basis. Everyone in our study reports drinking sugary beverages at least 3 times per week. Consuming sweet drinks is the primary focus of this study. We know it is really common, and we are trying to understand more about it. Our health coaches will help you make a goal about how many drinks you may want to reduce to, if that feels relevant to you.  We also think stress and cravings can play a big role in how much, or what we choose to drink.

*Start with a question for the participant to find out what their priorities are*

With that in mind, are there any particular concerns or changes you are wanting to make to the amount of sugary beverage you are drinking? *(e.g., how much sugar you consume, or to your beverage consumption more generally?)* What type of concerns do you have? What types of changes are you wanting to make? What interested you about this study?

**Rephrase and Summarize Goals**

e.g., I hear you want to drink less sugar; that is great, because our program can help with that.

- *Use the language participant uses, e.g., “overdrinking,” “soda cravings,” etc.*
- *If participant is focused on losing weight, redirect to ways of improving one’s health by focusing on behaviors that can be changed, such as making alternative SSB choices*
- *If participant very strongly resonates with problematic SSB behavior and triggers, etc., there is no need to probe too much, they get the idea!*
- *If participant is having difficulty identifying any SSB-related concerns, reassure participant that they don’t have to commit to a goal right now.*

*Program Summary/Check-In (about eating behavior)*

We hope that this program will help you take steps toward improving your health.

Well let’s end here. As a reminder we are scheduled to meet on _______ *(date/time).* I will send via email the link for the meeting. I will be sending you an informational document prior to the meeting that I will ask you to follow-along with during the session. I really look forward to working with you _______ (e*.g., next week*)!

*Note:* The call is to be performed in a private, confidential location. The idea of this call is to plan a seed and to get them thinking about what they might want to focus on in the session.

Provide reassurance that these types of SSB behaviors are very common. Coaches were instructed to take notes on participant answers to integrate them into personalized coaching sessions. Participants were required to provide a numeric response to emboldened questions during the introductory call.

#### Protocol for brief counseling session

*Program Background:*

This intervention is based on the Healthy Beverage Initiative sample (HBI) treatment model, in which a brief counseling session plus 2 booster phone calls contributed to a reduction in self-reported intake of sugar-sweetened beverages (SSB), especially for adults with overweight.

*Program Overview:*

- The counseling session is to be performed by a trained health coach and will last about 15 to 25 minutes.
- Training plan is as follows:
- Health coach will read through assigned “MI for health behaviors” materials, and discuss with supervisor and trainees in 6 weekly didactic trainings
- Health coach will first observe supervisor conduct the counseling session during the training sessions
- Health coach will practice counseling session script with other trainees in 3 to 4 weekly practice sessions (outside of weekly didactic training)
- Health coach will conduct the session, with supervisor observing/providing supervision remotely, as many times as necessary until health coach feels comfortable working independently
- Health coach will conduct the session independently, once supervisor has provided approval

*Goals of Counseling Session*:

1. Identify challenging SSB consumption patterns
2. Identify triggers for challenging SSB consumption
3. Collaborate on specific, achievable goal(s) for SSB consumption behavior change
4. Identify motivational factors to achieve goals
5. Identify modes of enlisting support for achieving goals and identify alternative healthy behaviors
6. Identify potential barriers towards achieving goals
7. Introduce a craving management/stress reduction tool
8. Rate self-efficacy (how confident is the pt in their ability to achieve their goal?)
9. Agree on next steps (booster calls, habit changes, etc.)

*Logistics prior to session:*

- Ensure that participant has received a confirmation email/calendar invite/link for counseling session appointment
- Review notes from introductory phone call
- Check whether participant has consented to being recorded
- Ensure you are performing an online video call from a private, quiet location
- Print/have access to:
- Counseling session script
- Psychoeducational Materials (for in-session)
- Type in your session notes and data points

*Logistics following session:*

- Email participant reminder for booster call 1 appointment (using email template) and add appointment to Sweet Study calendar (pt. initials, your name, day, time).
- This email will include overview of goals/importance/alternative behaviors, and link to craving management website.
- Ensure data has been updated with data from counseling session
- Let supervisor know, via email, the status of session, and whether recording has been saved. Schedule supervision session with supervisor and team once all the above tasks are completed.

#### Brief Counseling Session Script

***= Definitely ask this question*

*If unstarred, ask question(s) at your discretion, time allowing*

*Introduction and Assessment of SSB Consumption*

Great to speak with you, __________ (name of participant)! (*As you may remember*) My name is _____ from the Sweet Study.

Today’s discussion will be around 15 to 25 minutes. If it is okay with you, we’ll be recording this meeting for quality control and to make sure the staff are asking the best questions, as we are still training our staff. We’ll keep your information completely confidential. Any identifying information, like your name, will never be connected to any data we collect from you. Do you have any questions so far? Is it ok if I start the recording?

*(If participant consents, start recording)*

**First, we would like to thank you for your participation in The Sweet Study. As I mentioned on the phone, all participants in this study report drinking sugary beverages (*SSB’s; or can say “sugar sweetened beverages”*) at least 3 times per week. Consumption of sweet drinks is the primary focus of this study, and we are trying to understand more about it. That’s why I’ll be starting with asking you some specific questions about your intake.

**It would be helpful if you can walk me through what a normal day looks like for you with regard to sugary drinks. Sugary drinks, as a reminder, are beverages that have sugar added to them. Some examples include sodas, sweetened teas, sports drinks, energy drinks, smoothies and coffee drinks like frozen coffee drinks.

*(aim to get a specific # of total SSBs consumed per week, using the probes below, and confirm this number sounds accurate to participant)*.

Tell me about your workday; What are your favorite sweet  drinks, or the ones you have most often?:

What SSB’s do you typically have in the morning (e.g., sweetened teas and coffees): _________

How about Lunch (e.g., Iced Teas) _________

“ Afternoons/breaks _________

“ Nights (e.g., post-work cocktails) _________

How do your weekends/non-work days look with regard to sweet drinkss?____

*Summarize frequency and types of SSB consumption on a given week*

**** Thank you for these details! *So it sounds like you tend to have about XX SSBs on a work day, and XX beverages on a weekend/non work day…which totals to about XX drinks per week. Do I have that right?”*

**How do you feel about the current number of sweet drinks you have per week?(e.g., of 3 to 5 sodas per week)?

*Reflect/summarize/validate what client responds with.*

*Use open-ended questions, affirmations, reflections and summaries, as appropriate, to elicit change talk.*

**How interested are you in reducing the amount of sugary drinks you consume, o*n a scale from 0 (not at all interested) to 10 (most interested); in other words, how important would you say ______ is for you to ___ (e.g., reduce SSB intake) right now? ______ (write down number response)*

*What puts you at a ____ and not a 0 (or lower number than what they picked)?*

*What would it take for you to go from a ____ to a ____ (higher number)?*

*In other words, what might happen that would make the change more important?*

*Assessing concerns and interest in change (Focusing and evoking)*

**So you mentioned: ________________ (*e.g., you wish you drank less SSB’s).* What about having (*X drinks a day/a week)* concerns you? _________Tell me more about that.

(*re-iterate participant’s general SSB-related concerns; use their language*).

Tell me how you think that might relate to your health?

In your perfect world, what about __________ (*e.g., your relationship with SSB’s*) would be different?

In other words, how would you like for things to change, regarding __________ (*the number of sweet drinks you consume intake)?*

*Reflect/summarize/validate what client responds with. Use open-ended questions, affirmations, reflections and summaries, as appropriate, to elicit change talk.*

*(e.g., “It might be helpful to talk about your cravings for SSB’s at work, especially during times of high work stress;” or “So on the on the one hand, it could be helpful to you to discuss ____, but on the other hand, there might be some resistance to working on it right now)*

*(if not much change talk elicited, or participant doesn’t appear concerned, ask the following):*

What feels meaningful to you about the topic of sugary drink consumption? _________

How do others feel about your current SSB consumption? In other words, has anyone ever encouraged you to cut down? _________

Why? _________

What are the main topics that concern you, particularly as they relate to your health? _________

In what ways do you imagine your current level of SSB intake might relate to your current health? _________

*(If they are reluctant, find out what they do care about, sometimes it’s about family connections and not necessarily their own health directly. E.g., “My mother had diabetes and never met my children, and I worry about getting it myself. I care about living to see my grandchild grow up or get married”)*

What do you care about? _________

Focusing:

**We’re interested in the relationship between SSB intake and reducing risk for worsened overall health (like type 2 diabetes and heart disease)-- this is precisely the area of interest for our team. We’re eager to learn about sugary drinks directly from you. *On my agenda today is to provide you with information about sugar, particularly sugar in liquid form, and its links with our health, to identify some specific goals around your frequency of consumption of sugary beverages, and developing a plan to realistically achieve those goals. Let me know how that sounds to you.*

*Psychoeducation about high sugar consumption and its effects on health*

**One topic I briefly want to cover with you today is consuming sugar via sugary beverages. However, before I do, tell me what you already know regarding links between sugar or SSB intake and our health?

*Reflect/summarize/affirm.*

***Ask for permission*: Can I give you our overview of sugar? We’re really excited to share this information.

*If participant says yes, open up “Educational Materials_Sweet Study” powerpoint, and share your screen:*

**Great – I’m now going to share some visual information that I’d love to review with you. I’ll be highlighting the key points- please feel free to ask any questions along the way.

<Slide 1- ‘What’s the deal with sugar?>’ briefly review by highlighting the 3 main bullets

<Slide 2>:

**We used to think that too much added sugar was a problem because the empty calories caused weight gain. We now know that the problems go far beyond weight gain and obesity. Over time, too much sugar overloads our vital organs, which are not made to handle so much refined sugar at once. This overload actually prematurely ages our body due to all of the tax it puts on our organs. Even though you can’t see it, it sets us up for early liver disease, heart disease and diabetes. This happens even in people without overweight or obesity.

<Slide 3>

**So how much sugar is too much? The American Heart Association recommends limiting added sugar to anywhere from 6 to 9 teaspoons a day. That’s this much.

<Slide 4>

**However, when you look at how much sugar is in just one of these sugary drinks, you can see how easy it is to reach or even exceed that limit with just one drink. You mentioned you like to drink _____- which has ____ of sugar per serving. I also want to highlight that while 12oz servings are represented in this slide, it is important to note that many of these drinks are typically consumed in larger sizes, and therefore have even more sugar.  For instance, Gatorade tends to be consumed in the 20 ounce size.

<Still on Slide 4>

**Now let’s compare this amount of sugar in ___ drinks (frequency pt says they drink) to the recommended limit for the entire day. What are your reactions to that discrepancy?

<Slide 5>

**Now that you’ve seen how much sugar is in these drinks, you may not be surprised that the average American consumes 17 to 19 teaspoons of added sugar per day.

**It’s at these levels that the serious, life-threatening diseases we discussed earlier can occur, like liver disease and heart disease.

<Slide 6>

**Many of us are hooked on sugar and it’s not uncommon. But we can take control over it!

So what can we do? The easiest way to reduce your sugar consumption is by reducing or abstaining from sugary drinks.  *What are your thoughts on these replacement beverages?*

<Slide 7>

**Alternatively, we can gradually limit our sugar consumption by diluting our preferred SSBs. What are your thoughts on this idea?

<Slides 8 and 9 are optional pieces to review>

<End slideshow>

** I know this is a lot of information I’m presenting you. What do you think about all of this? How does this affect your feelings about your current level of sweet drinks you consume?

*If they express concerns about how much they are drinking*: That is common. Many people exceed the healthy limit.

*>(If they are reluctant/defensive, this is another chance to ask about overall caring/concerns.)*

**How would you feel about turning now to some goal-setting around drinking sweet beverages?

*Planning*

*Note to interviewer: Have participant identify ONE realistic goal, based on the problematic SSB behavior they have identified above. Take a few minutes to explore what might be feasible for them over the next week.*

*Have them write this down on a piece of paper, or type it into notes section of their powerpoint.*

*Guide them to respond to the additional questions regarding importance of goal and barriers to goal.*

<Slide 10: What’s your primary goal?>

**Based on what you’ve shared with me earlier regarding frequency of SSB intake, what feels like a good realistic goal for you over just the next week? ____ *In other words, what would you like your SSB intake to look like throughout the week?*

**(*reflect back identified goal)* Ok great, and so now let’s talk about how you can go from ___ SSB’s to ___ SSBs per day/perweek…

Explore alternative drinks:

**What ideas have you already thought about regarding how you could manage challenging situations (use any examples provided by pt), when you might be tempted to drink? _________

**For instance, what might be an alternative drink that you can choose, that may be healthier? *(return to slide on alternative beverages in SSB powerpoint if needed)*

<Slides 6 and 7 of SSB ppt>

*(if relevant)* Here are a few options that our team has come up with. How do any of these options look to you?

(If participant chooses “diet soda”)

While diet soda has less/no sugar compared to regular soda, a small amount of research suggests that drinks with artificial sweeteners may not be helpful for reducing weight or insulin resistance (we have included some resources on this topic in your educational materials PDF). We want to encourage you to choose drinks that are naturally unsweetened (like water).

*Consider asking the following questions, as relevant:*

*What else could help you reach you goal of consuming ___ SSBs?*

*How realistic do you think it is to decrease ______ (e.g., SSB intake) from _____ to ______?*

*Which days do you think you can ______ (e.g., reduce SSBs)?*

*Re-check goal:  Confidence and Calibration; Assessing and dealing with potential barriers*

***Now that we have identified some new ideas, how confident are you that you can meet your goal of ______? (reiterate pt’s goal/plan around SSB intake)*

***Please pick the number 1-10, 1 being low confidence to meet your goal and 10 being extreme confidence to meet your goal*

*<participant picks number; make sure to have them decide on 1 whole number (not halves)>*

If it’s a low rating: do more troubleshooting. Assess barriers. Perhaps make goal easier, and reassess self-efficacy.

***What makes it a ____ and not a _____ (lower number)? What would it take to make your rating go from a ___ to a _____ (1 to 2 points higher)?*

If it’s a high rating: *Fantastic.  Your self-confidence tells me that you are going to be successful. Of course, there will be slips sometimes but you will be making a very meaningful change.*

*<Slide 11>*

*** What are the primary reasons for this goal? In other words, what makes it important to you? Guide pt to write this down.*

*<Slide 12>*

***Over the next week, what obstacles do you anticipate? Let’s talk through ways to overcome these obstacles. Guide pt to write this down.*

*Household Situation: if they live with others (OPTIONAL)*

*Tell me a bit about who lives at home, and whether anyone drinks SSBS at home*

*(if it’s a potential challenge) How do you think people at home will respond to you trying to limit sugary drinks?*

*Who could help support you in your new goal? _________*

*How can you enlist their help? _________*

*(Guide participant to write this down on pamphlet under stumbling blocks)*

*We will be giving you materials today to take home with you and share with your family or roommates. While you cannot control others’ choices, you can offer the information so that they can help support you in reaching your goals.*

*How would you feel about sharing these materials with your (family/roommate)?*

*Our experience tells us that having support from others with whom you live and spend time makes us much more successful at reaching our health goals.*

*Wrap-up and introduction to craving management online tools:*

*** Congratulations on your bold new steps toward better health. It is not easy to identify triggers for our _____ (SSB intake) and come up with specific goals in such a short period of time, but you’ve really gotten the ball rolling!*

***We have created some tools to help you reach your goals of reducing the amount of sweet drinks you are consuming. I will email you a link to these tools- which help with managing cravings for SSBs or to help with managing stress, which are both very common triggers for turning to sugary beverages. These are 3-8 minute audio modules that we want to encourage you to listen to at times you feel it could benefit you in reducing sugary beverages. I’ll check in with you about these tools and how they’re helpful to you at our next call. Our hope is that you can use these in your daily life.*

***I’d like to connect again in one week to see how things are going with your goals. I want to hear how you are doing no matter what; My role is to support you in your goals, and if you don't meet your goals, I want to hear about that too, okay? We can absolutely talk you through any road blocks you might run into. Running into barriers is common and normal, so please be open and honest about any challenges you might be facing! We want the calls to be helpful for you.*

***Schedule a time for first booster phone call/ check-in (5-10 minutes)-- should be 1 week from this session.*

*Date/Time: _________________________________*

*I will call you next week on _________ . What is the best number? Alternatively, we can meet via video if you prefer.*

***Thanks so much for your time. You are working towards a very meaningful change in your life. You are also contributing to an important study that will tell us if changing what we drink at work, and at home, can improve our health. Thank you for your time and important contributions.*

***Interviewer to email participant their list of stated goals for program, calendar invite for next booster call, and tips on stress management and craving management.*

**MAKE SURE TO STOP RECORDING**

#### Counselor notes for documentation after coaching call

- Document primary focus for session
- Document primary triggers for the identified consumption behavior
- Document any significant notes from phone contact.
- Identify key challenging drinking patterns (e.g., beverage types, beverage amounts, context
- of drinking):
- Identify triggers for challenging drinking
- Describe frequency and nature of drinking
- Document participant motivational factors and values
- Describe specific, achievable goal(s) for drinking (over the next week)
- Importance Scale Rating (1 – 10) and why (describe):
- Identify potential barriers and vulnerabilities (stumbling blocks)
- Plans for enlisting support & alternative behaviors
- Self-Efficacy Scale Rating (1 – 10) and describe
- Additional notes:

### Appendix 2: audio module scripts and samples of the study website

Stress Management for Sugar/SSB Cravings

(3 minute script)

*Follow Stress Management Script, speaking slowly and attending to the pace of the participant.*

*Stress Management Exercise*

- Welcome!  You might sit up with a straight, easy posture, keeping your eyes open and softly focused on something in front of you. Find a comfortable position where you feel fully supported. Allow your hands to fall comfortably in your lap. Simply allow your body to become still. Allow your shoulders, chest, and stomach to relax... (*Pause*)…
- Allow your attention to be focused on your breath and away from the noise, the thoughts, the feelings, the concerns that may usually fill your mind… (*Pause*)…
- Begin by taking two or three deeper breaths, letting the air flow all the way into your belly, without any push or strain, and then flow gently back out again… (*Pause*)…  on the next out breath, allow your gaze to soften or close your eyes completely…(*pause*)
- Now let your breathing find its own natural, comfortable rhythm and depth, and gently focus your attention on the feeling of your breath as it comes in at the tip of your nose, moves through the back of your throat, into your  lungs, and back out again, letting your stomach  move out and in naturally with each breath... breathing into your belly…….calm, deep belly breathing (*Pause*)…
- Now, focus on the sensation of breath as it comes in and out of your nostrils, You may notice that the breath is cooler coming in, warmer flowing out*…(Pause)….*
- As you continue, you will notice that the mind gets caught up with thoughts,. You may find yourself remembering something from the past, thinking about the future, or flowing into fantasy. This is to be expected. This is the nature of the mind… (*Pause*)…
- When you notice this—without self-judgment--gently return your attention to the breath. Simply let your body feel the flow of breath, as it moves in and out, in and out, freely shifting with a gentle, natural rhythm*… (Long Pause)…*
- You may also notice emotions arising —see if you can label them.  You might feel a sense of anxiety, sadness, anger, joy, or peace. Allow whatever is there to be there, as you remain observing – simply label them instead of getting swept up in them, allowing them to run the show. Notice how the feelings shift and change, and then return your awareness to the centeredness of the breath*... (Pause)…*
- Stay in the present moment… (*Long Pause*)…
- Notice any body sensations. Check in to see how hungry or full you may feel right now……  If feelings of thirst or hunger are present, ask yourself, Are these feelings of hunger or thirst from true need, or from boredom, sadness or stress?
- Simply ride with the flow of breath, feeling it move in and out, freely shifting with a gentle, natural rhythm… (*Pause*)…
- And now gently bring your attention back just to the breath… (*Pause*)…
- bring your attention back into your body
- Move your body around gently to promote comfort and groundedness where you are sitting..  Can you let your body feel ease? *… (Pause)…*
- When you are ready, open your eyes and gently stretch out.
- You’ve just finished a ‘mini-meditation’, This technique can help excess stress and tension dissipate, and, you can use this anywhere and at any time.

*Reflections:*

- See if you can allow this feeling of being grounded and steady stay with you as you move through your day
- Before you log off, please take a moment to rate your current level of craving for sugary drinks, as well as your current level of stress, Great work today!

Stress Management for Sugar/SSB Cravings

(5 minute script)

*Follow Stress Management Script, speaking slowly and attending to the pace of the participant.*

*Introduction*

- A brief check-in is a powerful way to stay calm and centered throughout the day
- This involves taking several slow breaths, letting go of any tension in the body, and becoming aware of the body, the emotions, and the thoughts.
- When you are alone, you can close your eyes to do this practice. This practice can also be done with your eyes open when around others.  It is a great way to calm and ground yourself, and because it is an internally guided process, nobody will even know you’re doing a check in.
- It’s particularly useful to use this practice right before drinking a sugary beverage because you can become more aware of why you are drinking. Are you thirsty, hungry, or feeling a craving for sugar?
- *Now let’s dive in to our stress management exercise.*

*Stress Management Exercise*

- You might sit up with a straight, easy posture, keeping your eyes open and softly focused on something in front of you. Find a comfortable position where you feel fully supported. Allow your hands to fall comfortably to your lap. Simply allow your body to become still. Allow your shoulders, chest, and stomach to relax... (*Pause*)…
- Allow your attention to be focused on your breath and away from the noise, the thoughts, the feelings, the concerns that may usually fill your mind… (*Pause*)…
- Begin by taking two or three deeper breaths, letting the air flow all the way into your belly, without any push or strain, and then flow gently back out again… (*Pause*)…  on the next out breath, allow your gaze to soften or close your eyes completely …(*pause*)
- Now let your breathing find its own natural, comfortable rhythm and depth, and gently focus your attention on the feeling of your breath as it comes in at the tip of your nose, moves through the back of your throat, into your lungs, and back out again, letting your stomach move in and out naturally with each breath... breathing into your belly…….calm, deep belly breathing (*Pause*)…
- Now, focus on the sensation of breath as it comes in and out of your nostrils, You may notice that the breath is cooler coming in, warmer flowing out*… (Pause)….*
- As you continue, you will notice that the mind gets caught up with thoughts. You may find yourself remembering something from the past, thinking about the future, or flowing into fantasy. This is to be expected. This is the nature of the mind… (*Pause*)
- When you notice this—without self-judgment--gently return your attention to the breath. Simply let your body feel the flow of breath, as it moves in and out, in and out, freely shifting with a gentle, natural rhythm… (*Long Pause*)…
- You also notice emotions arising —see if you can label them.  You might feel a sense of anxiety, sadness, anger, joy, or peace. Allow whatever is there to be there, as you remain observing, – simply label them instead of getting swept up in them, allowing them to run the show. Notice how the feelings shift and change, and then return your awareness to the centeredness of the breath... (Pause)…
- Stay in the present moment… (*Long Pause*)…
- And now again let your mind relax and let go.  Return your attention to your breathing, secure in the knowledge that you are using mindful awareness to understand yourself and your drinking patterns, secure in the knowledge that you are putting together the pieces of the puzzle that will lead you to take greater control of your life and your drinking. … (*Pause*)…
- Check in to see how hungry or full you may feel right now.  Are feelings of hunger or thirst from true need, or from boredom, sadness or stress?
- How might you attend to some of these feelings in a healthier way, without turning to sugary beverages?....If you’re noticing stress, could you take a walk? …If you’re noticing anger, could you talk to a friend?....Think about how you might manage these emotions effectively….(long pause)
- Simply ride with the flow of breath, feeling it move in and out, freely shifting with a gentle, natural rhythm… (*Pause*)…
- And now gently bring your attention back just to the breath… (*Pause*)…
- bring your attention back into your body *… (Pause)…*
- Move your body around gently to promote comfort and groundedness where you are sitting.  You can let your body feel at ease.
- When you are ready, open your eyes and gently stretch out.
- You’ve just finished a ‘mini-meditation’, This technique can help excess stress and tension dissipate, and, you can use this anywhere and at any time.

*Reflections:*

- Let this feeling of being grounded and steady stay with you as you go through your day, Thank yourself for taking this time to soothe feelings of stress and craving.
- Before you log off, Please take a moment to rate your current level of craving for sugary drinks, as well as your current level of stress, Great work today!

Stress Management for Sugar/SSB Cravings

(5 minute script)

*Follow Stress Management Script, speaking slowly and attending to the pace of the participant.*

*Introduction*

- A brief check-in is a powerful way to stay calm and centered throughout the day
- This involves taking several slow breaths, letting go of any tension in the body, and becoming aware of the body, the emotions, and the thoughts.
- When you are alone, you can close your eyes to do this practice. This practice can also be done with your eyes open when around others.  It is a great way to calm and ground yourself, and because it is an internally guided process, nobody will even know you’re doing a check in.
- It’s particularly useful to use this practice right before drinking a sugary beverage because you can become more aware of why you are drinking. Are you thirsty, hungry, or feeling a craving for sugar?
- *Now let’s dive in to our stress management exercise.*

*Stress Management Exercise*

- You might sit up with a straight, easy posture, keeping your eyes open and softly focused on something in front of you. Find a comfortable position where you feel fully supported. Allow your hands to fall comfortably to your lap. Simply allow your body to become still. Allow your shoulders, chest, and stomach to relax... (*Pause*)…
- Allow your attention to be focused on your breath and away from the noise, the thoughts, the feelings, the concerns that may usually fill your mind… (*Pause*)…
- Begin by taking two or three deeper breaths, letting the air flow all the way into your belly, without any push or strain, and then flow gently back out again… (*Pause*)…  on the next out breath, allow your gaze to soften or close your eyes completely …(*pause*)
- Now let your breathing find its own natural, comfortable rhythm and depth, and gently focus your attention on the feeling of your breath as it comes in at the tip of your nose, moves through the back of your throat, into your lungs, and back out again, letting your stomach move in and out naturally with each breath... breathing into your belly…….calm, deep belly breathing (*Pause*)…
- Now, focus on the sensation of breath as it comes in and out of your nostrils, You may notice that the breath is cooler coming in, warmer flowing out*… (Pause)….*
- As you continue, you will notice that the mind gets caught up with thoughts. You may find yourself remembering something from the past, thinking about the future, or flowing into fantasy. This is to be expected. This is the nature of the mind… (*Pause*)
- When you notice this—without self-judgment--gently return your attention to the breath. Simply let your body feel the flow of breath, as it moves in and out, in and out, freely shifting with a gentle, natural rhythm… (*Long Pause*)…
- You also notice emotions arising —see if you can label them.  You might feel a sense of anxiety, sadness, anger, joy, or peace. Allow whatever is there to be there, as you remain observing, – simply label them instead of getting swept up in them, allowing them to run the show. Notice how the feelings shift and change, and then return your awareness to the centeredness of the breath... (Pause)…
- Stay in the present moment… (*Long Pause*)…
- And now again let your mind relax and let go.  Return your attention to your breathing, secure in the knowledge that you are using mindful awareness to understand yourself and your drinking patterns, secure in the knowledge that you are putting together the pieces of the puzzle that will lead you to take greater control of your life and your drinking. … (*Pause*)…
- Check in to see how hungry or full you may feel right now.  Are feelings of hunger or thirst from true need, or from boredom, sadness or stress?
- How might you attend to some of these feelings in a healthier way, without turning to sugary beverages?....If you’re noticing stress, could you take a walk? …If you’re noticing anger, could you talk to a friend?....Think about how you might manage these emotions effectively….(long pause)
- Simply ride with the flow of breath, feeling it move in and out, freely shifting with a gentle, natural rhythm… (*Pause*)…
- And now gently bring your attention back just to the breath… *(Pause)…*
- bring your attention back into your body … *(Pause)…*
- Move your body around gently to promote comfort and groundedness where you are sitting.  You can let your body feel at ease.
- When you are ready, open your eyes and gently stretch out.
- You’ve just finished a ‘mini-meditation’, This technique can help excess stress and tension dissipate, and, you can use this anywhere and at any time.

*Reflections:*

- Let this feeling of being grounded and steady stay with you as you go through your day, Thank yourself for taking this time to soothe feelings of stress and craving.
- Before you log off, Please take a moment to rate your current level of craving for sugary drinks, as well as your current level of stress, Great work today**!**

Craving Management (Urge Surfing) for Sugar/SSBs

(5 minute introductory script)

*Follow the Urge Surfing Script, speaking slowly and consistently paced.*

*Introduction:*

- Welcome. Today, I will lead you through a brief practice to help you with cravings for sugary beverages in a powerful way, right at the moment they arise.
- You can use this exercise when you notice a craving. A craving usually feels like a strong, irresistible urge for something, like a sugary beverage.
- Cravings can be really sneaky, and before we know it, we’re completely helpless and under their control.
- Think of managing a craving like staying on a surfboard. How cravings arise is pretty similar to surfing a wave:  Waves come up, and if we’re not looking out for them, and don’t know how to surf them, they knock us off balance and suck us into the undertow.
- The more we see that cravings are just sensations in our body taking us for a ride along the waves, the more we can learn to ride them out to shore and not give in to them.
- Being more mindful keeps you from being sucked into the craving or crashing into the wave. You are getting a new surfboard today.
- With a craving, first, you need to RECOGNIZE that the wave is coming and then RELAX into it.
- Relaxation and Acceptance are the key to learning how to ride the waves of craving. If you are too tense, you can actually get thrown from your surfboard.
- See if you can ACCEPT or ALLOW this wave to come on just as it is, without trying to ignore the craving or distract yourself from it.
- A helpful way to “ride the wave” that arises from craving is to INVESTIGATE IT – become interested in what the wave feels like, noticing sensations, emotions and thoughts that come along with this craving. Simply notice and label the sensations, emotions or thoughts that arise in the moment. This helps you to become less identified or wrapped up in what you are feeling by making you more of a wise observer or witness of what is arising. For  example, if you notice the sensations of burning or tightness in your stomach, you can simply say to yourself, “burning, burning…..tightness, tightness.”. Just keep describing what the sensations feel like as the craving builds, crests and then subsides. Mentally NOTE inside your head or out loud what is going on from moment to moment.
- Using these techniques, you can ride your cravings out all the way to the shore.
- If you still find the craving overwhelming by the end of this exercise, try another method like going for a brisk walk, or drinking water. Through observation of the craving cycle over time, you will come to observe and Trust that craving will eventually subside.

*Craving Management/Urge Surfing Activity*

- Now I will walk you through the craving management exercise.
- First, go and get a tasty, sugary beverage that you prefer. As a reminder, this should be something that is super rewarding or craveable for you, like a soda or juice.
- Feel free to hit the pause button while you find a drink
- Hold the beverage in your hand and just notice how this can bring on a craving or urge to drink.
- First, RECOGNIZE that the wanting or craving will be coming on and RELAX into it.
- Rather than trying to resist the craving by gritting your teeth, just let go and feel it come on. Notice the urge to distract yourself or try to do something about the craving. And instead, ALLOW and ACCEPT this wave of craving as it is.   You have no control over it anyway. What you can control is how you meet it. Letting go and riding the wave is the only thing that can lessen the intensity. So let go, and surf the wave.
- To catch the wave of wanting, you have to study it carefully, to investigate it. Be curious. Ask “what is going on in my body right now?”  See what arises, letting it come to you naturally.
- Where does the craving start in your body? Where do you feel it the most?
- What does it really feel like? Is it tightness in your chest?  Is it a burning feeling in your stomach?  Is it a mouth-watering sensation?
- How strong is the craving? On a 1 to 10 scale, with 1 being not at all and 10 being the strongest, where do you fall?
- Finally, NOTE the experience using single words or phrases. This keeps you here now, focused and riding the wave.
- What does it feel like as the craving comes on and peaks? You might note: clenching, rising, burning, heat, restlessness, anxiety…. What do you feel as the craving subsides?……perhaps noting vibration, tightness, tingling, lessening, relaxing, relief,
- The more you can note your own experience moment by moment, the more likely you will be able to ride the wave of craving to its end.
- Follow the wave until it completely subsides. If you get distracted, or your mind shifts to something else, that’s okay. Simply return to being curious and ask: “what is going on in my body right now?”
- Ride the craving until it is completely gone.  Ride it all the way to shore.
- When you are done surfing the urge to drink, take a moment to thank yourself for taking this time for yourself and being willing to do something different with your urge to drink sugary beverages. This same exercise works with food cravings, so you may try it in that context as well. you may also set an intention for the next few minutes, hour, or day with regard to your craving. The intention could be as simple as returning to this same practice the next time you feel a craving.
- Let yourself breathe gently, slowly, and naturally, and let your body feel the ease of just breathing, and being.

*Reflections/Wrap-up:*

- That’s it! take a moment to reflect on your experience with this urge surfing exercise. Not so bad was it? You’ve got this! With practice craving management gets easier and you may discover that you are an excellent surfer.
- You can practice this technique in two ways:
- Whenever you notice yourself having an urge to drink tempting sugary beverages…or whenever you notice urges to go back to other old habits you are trying to break.
- You can also practice this on a regular basis by setting aside time to use this technique. Through this kind of routine practice, you can get better at urge surfing during the times when you really need it.
- With practice, urges become easier to ride out. You may even start to feel a sense of pride or accomplishment as you successfully surf urges and act according to your values, instead of according to your urges.
- Before you log off, Please take a moment to rate your current level of cravings and thirst, once this audio session has ended. Great work today! Keep it up.

## Appendix 3: sample education material

### Appendix 4: Training program syllabus

#### Motivational Interviewing Health Coach Training Plan

**Week 1: Overview of HBI Program & Introduction to Motivational Interviewing**

All health coaches in the Sweet Study completed six weeks of motivational interviewing (MI) didactic training over Zoom with a licensed clinical psychologist trained in MI. Sessions involved watching MI video clips, role-playing with partners, and discussing readings from Rollnick and Miller.^1^ Session, one introduced an MI-informed approach of directive, patient-centered counseling. Trainees discussed readings assigned from chapters 1-3 of Miller and Rollnick. Discussion centered around questions such as *“What is the spirit of MI?”* and *“What is unique to MI?”* Trainees were introduced to the spirit of MI including partnership, acceptance, absolute worth, and accurate empathy. The four core components of MI were introduced including engaging, focusing, evoking, and planning. In addition, the group explored and resolved ambivalence towards behavioral change using examples from patients with substance use disorder. Trainees were also presented with basic information such as the study timeline, information regarding participant confidentiality and consent, and the Motivational Interviewing Treatment Integrity (MITI) coding manual.^2^

**Week 2: Using MI framework to Engage participants**

Session two introduced using the MI framework to engage participants pulling material from chapters 4-7. Week two presented using MI to address sugar intake and health issues and began role playing the phone call component of the intervention sessions. This session introduced trainees to topics, such as engaging versus disengaging questions, listening and understanding dilemmas, core interview skills, and exploring values. After discussing early engagement and rapport-building factors, trainers introduced forms of feedback and reflections. Trainees were given direction on the length of reflection as well as direction in reflection. After practicing building engagement and providing reflections, participants explored how to evoke client values using open-ended interview techniques to derive participant values such as: *What matters most to you, Why is ____ important to you, and How do you express values in your life.* Lastly, trainees synthesized all the information from this session and conducted role-playing from case vignettes.

**Week 3: MI- Focusing**

Week three reviewed chapters 8-11on focusing. In addition, facilitators showed the video clip on engaging and agenda mapping: *Living with Diabetes*. Participants continued practicing role plays using phone call scripts and rated each other using MITI coding. Focusing is a prerequisite for the evoking and planning stages. The facilitators presented various focusing scenarios such as when a client may not have a clear source of focus, when there may be several options, or when the focus may be clear. The group explored sources of focus (see Table 1) as well as how to search for strengths and openings for change when motivating a client. Facilitators discussed common traps such as assuming expertise, rectifying gaps in knowledge, and directly telling clients what to do which may lead to clients presenting with defensiveness or not following the health coach’s direction. Good practices at this stage of MI include following the elicit-provide-elicit (E-P-E) framework by asking for permission to share knowledge, providing information, and eliciting the client’s interpretation. Lastly, trainees paired up and practiced engaging and then focusing clients using the phone coaching script.

Three Sources of Focus

| The client | “My blood sugar is running too high, and I want to adjust my diet” |
| --- | --- |
| The setting | A weight loss clinic: “My wife says I need to eat better” |
| Clinical Expertise | Clinical expertise e.g., a nurse monitoring medication for a patient with AIDS wants to discuss nutrition |

*Note:* The three sources of focusing were presented alongside three styles of focusing: 1) directing, where the provider determines the focus 2) following, where the client directs the focus, and 3) guiding, where there is a collaborative search for direction.

**A4. Week 4: MI-Evoking**

In the fourth MI training session, trainees practiced evoking clients through role-playing intervention components, pulling from chapters 12-18.^1^ In this session, trainees focused more on ways to evoke change talk and motivate clients to move towards a resolution of ambivalence and towards change. Trainees distinguished preparatory versus mobilizing change-talk. The DARN acronym provided a way to instigate preparatory change talk, speaking to desire, ability, reasons, and need that one would want to evoke change. The CATS acronym offered a mnemonic for signals that change may be occurring including commitment, activation, and taking tangible steps towards change. The facilitators asked trainees to distinguish change talk with its ambivalent counterpart, sustain talk. Sustain talk involves any speech that can be uttered on behalf of change can also be spoken as an equal and opposite reaction on behalf of the status quo (see table 5 for a delineation between change and sustain talk). Trainees practiced responding to discord that may arise with change talk by synthesizing change and sustain talk that may arise in each scenario and providing double-edged reflections that point out dialectics that exist for clients. Facilitators then introduced the topic of developing discrepancy between a present and

**Week 5: MI- Planning**

The last session of MI training covered the topic of planning and pulled content from chapters 19-22.^1^ In this session, worked on developing change plans and practiced making specific change plans versus vague plans. When mapping specificity, trainees were taught to confirm goals with clients and develop specific plans of action with a statement of intent such as, “I want to lose 5 lbs. this month.” Trainees learned about ways to enhance commitment by encouraging clients to draw from social support systems and utilize self-monitoring between coaching sessions. Lastly, trainees practiced planning the bridge to change in pairs and worked through intervention materials that implement the planning stage of MI.

**Week 6: Ongoing Supervision**

Throughout the course of the intervention, health coaches met weekly in groups with licensed clinical supervisors to discuss progress with participants and continue to learn MI techniques. Before each session, health coaches were asked to provide specific participant IDs that they would like to review with the supervisor in the group setting. The supervisor would lead coaches through role-plays and explore response styles that may have been more validating, evocative, and may have promoted more change talk. In addition, supervisors provided specific feedback on how participants could improve their coaching style to adhere to the MI principles and score more highly on the MITI coding system.^2^

Brief MI Intervention Practice Tracker Log

| Practice | Date(s) or week(s) completed | Comments, experiences, Reflections, insights, leading and receiving.  What did you learn? How did the other person respond? |
| --- | --- | --- |
| Phone Call (~30 min per person) | *****Minimum of 2 practices required | Leading:  Receiving: |
| Counseling Session (~30 min per person) | *Minimum of 3 practices required | Leading:  Receiving: |
| Booster Call (~15 min per person) | *Minimum of 2 practices required | Leading:  Receiving: |

*Note:* Coaches were asked to review session scripts before practice sessions with other students; they took on a receiving or leading role based on who was role-playing the participant and health coach. Emailing a completed practice log to training coordinator ensured readiness to serve as a health coach for study. The supervisors did not use an MI coding form to code fidelity to MI to serve as a health coach. However, MITI 4^2^ coding was used for most recorded sessions throughout the intervention to provide feedback and supervision.

## Appendix

### Table S1: Additional MITI Fidelity to MI principles (N=41)

| Behavior count | Mean (SD) | Median | Intraclass correlation |
| --- | --- | --- | --- |
| Giving Information | 7(3.55) | 6.00 | .604 |
| Persuade | .80(.92) | 1 | .620 |
| Persuade with Permission | .61(.77) | 0 | .067 |
| Question | 16.37(5.49) | 16 | .969*** |
| Simple reflection | 6(2.36) | 6 | .499 |
| Complex reflection | 4.29(2.47) | 4 | .846* |
| Affirm | 1.68(1.36) | 1 | .947*** |
| Seek collaboration | 1.41(.99) | 1 | .792* |
| Emphasize autonomy | 0.27(0.50) | 0 | .774* |
| Confront | .02(.15) | 0 | - |
| *ICC N=8 | | | |
